# Effects of Different Nitrogen Sources and Ratios to Carbon on Larval Development and Bioconversion Efficiency in Food Waste Treatment by Black Soldier Fly Larvae (*Hermetia illucens*)

**DOI:** 10.3390/insects12060507

**Published:** 2021-05-31

**Authors:** Yan Lu, Shouyu Zhang, Shibo Sun, Minghuo Wu, Yongming Bao, Huiyan Tong, Miaomiao Ren, Ning Jin, Jianqiang Xu, Hao Zhou, Weiping Xu

**Affiliations:** 1 Department of Environmental Ecological Engineering, School of Marine Science and Technology, Dalian University of Technology, Panjin 124221, China; lyllyying@mail.dlut.edu.cn (Y.L.); Zshouyu@mail.dlut.edu.cn (S.Z.); wumh@dlut.edu.cn (M.W.); biosci@dlut.edu.cn (Y.B.); tonghuiyan@dlut.edu.cn (H.T.); renmiao18@mail.dlut.edu.cn (M.R.); 18342782101@163.com (N.J.); zhouhao@dlut.edu.cn (H.Z.); 2 Panjin Institute of Industrial Technology, Dalian University of Technology, Panjin 124221, China; Jianqiang.Xu@dlut.edu.cn; 3 School of Life Science and Pharmaceutical Sciences, Dalian University of Technology, Panjin 124221, China; sunshibo@mail.dlut.edu.cn; 4 Key Laboratory of Industrial Ecology and Environmental Engineering, Dalian University of Technology, Dalian 116024, China

**Keywords:** nitrogen source, carbon to nitrogen ratio, food waste, urea, black soldier fly larvae, *Hermetia illucens*

## Abstract

**Simple Summary:**

Black soldier fly larvae (BSFL) have received global research interest and industrial application due to their high performance on the organic waste treatment. However, the substrate C/N property, which may affect larvae development and the waste bioconversion process greatly, is significantly less studied. The current study focused on the food waste treatment by BSFL, compared the nitrogen supplying effects of 9 nitrogen species (i.e., NH_4_Cl, NaNO_3_, urea, uric acid, Gly, L-Glu, L-Glu:L-Asp (1:1, *w*/*w*), soybean flour, and fish meal), and further examined the C/N effects on the larval development and bioconversion process. We found that NH_4_Cl and NaNO_3_ led to poor larval growth and survival, while 7 organic nitrogen species exerted no harm to the larvae. Urea was further chosen to adjust the C/Ns. Results showed that lowering the C/N from the initial 21:1 to 18:1–14:1 improved the waste reduction and larvae production performance, and C/N of 18:1–16:1 was further beneficial for the larval protein and lipid bioconversion, whereas C/N of 12:1–10:1 resulted in a significant performance decline. Therefore, the C/N range of 18:1–16:1 is likely the optimal condition for food waste treatment by BSFL and adjusting food waste C/N with urea could be a practical method for the performance improvement.

**Abstract:**

Biowaste treatment by black soldier fly larvae (BSFL, *Hermetia illucens*) has received global research interest and growing industrial application. Larvae farming conditions, such as temperature, pH, and moisture, have been critically examined. However, the substrate carbon to nitrogen ratio (C/N), one of the key parameters that may affect larval survival and bioconversion efficiency, is significantly less studied. The current study aimed to compare the nitrogen supplying effects of 9 nitrogen species (i.e., NH_4_Cl, NaNO_3_, urea, uric acid, Gly, L-Glu, L-Glu:L-Asp (1:1, *w*/*w*), soybean flour, and fish meal) during food waste larval treatment, and further examine the C/N effects on the larval development and bioconversion process, using the C/N adjustment with urea from the initial 21:1 to 18:1, 16:1, 14:1, 12:1, and 10:1, respectively. The food wastes were supplied with the same amount of nitrogen element (1 g N/100 g dry wt) in the nitrogen source trial and different amount of urea in the C/N adjustment trial following larvae treatment. The results showed that NH_4_Cl and NaNO_3_ caused significant harmful impacts on the larval survival and bioconversion process, while the 7 organic nitrogen species resulted in no significant negative effect. Further adjustment of C/N with urea showed that the C/N range between 18:1 and 14:1 was optimal for a high waste reduction performance (73.5–84.8%, *p* < 0.001) and a high larvae yield (25.3–26.6%, *p* = 0.015), while the C/N range of 18:1 to 16:1 was further optimal for an efficient larval protein yield (10.1–11.1%, *p* = 0.003) and lipid yield (7.6–8.1%, *p* = 0.002). The adjustment of C/N influenced the activity of antioxidant enzymes, such as superoxide dismutase (SOD, *p* = 0.015), whereas exerted no obvious impact on the larval amino acid composition. Altogether, organic nitrogen is more suitable than NH_4_Cl and NaNO_3_ as the nitrogen amendment during larval food waste treatment, addition of small amounts of urea, targeting C/N of 18:1–14:1, would improve the waste reduction performance, and application of C/N at 18:1–16:1 would facilitate the larval protein and lipid bioconversion process.

## 1. Introduction

Larvae of black soldier fly (BSFL), Diptera:Stratiomyidae, *Hermetia illucens* (Linnaeus), are capable of converting various biowaste into protein-rich insect biomass and nitrogen-rich organic fertilizer [1,2,3] and have received worldwide research interest and fast-growing industrial application [4,5]. The application of BSFL for waste processing has expanded from tropical and temperate countries [4,5] to Russia [6], Canada [7], and Near East Turkey [8] in recent years. Amongst all the parameters that may impact the larval development and biowaste conversion process, ones such as temperature, moisture content, and pH are critically analyzed [9,10,11]. In contrast, the substrate C/N property, which may play a crucial role in the larval development and bioconversion process, is largely less studied. Several studies have examined the C/N effects indirectly, for instance, Bessigamukama et al. [12], Ewald et al. [13], and Lopes et al. [14] have added different amounts of biochar [12], fish [13], or mussel [14] to the grain or bread substrate for BSFL treatment, and Pang et al. [15] has studied C/N effects on the greenhouse gas emission; however, the C/N effects on larval development are still not clear and the optimal C/N range for a high bioconversion performance has not been achieved, since the substrate total C/N ratios were not determined or the bioconversion efficiencies of BSFL treatment were not examined in those studies.

The BSFL are able to degrade a wide range of organic waste, such as animal manure, food waste, abattoir waste, and aquaculture waste [16,17,18,19]. These wastes have different C/N properties, and BSFL are found to be largely more adapted to the C/N range of <20:1 and less suitable to the C/N range of >20:1. For instance, poultry, swine, and human feces, typically having C/N of 9:1 to 15:1, were found to be well-degraded by BSFL, while cow manure, having C/N of 20:1 to 30:1, was less decomposed by BSFL [20,21]. Poultry feed (C/N 18:1), food waste (C/N 14:1), and abattoir waste (C/N 6:1) showed acceptable and comparable decomposition, while fruit and vegetable waste (C/N 24:1) was found to be less processed by the BSFL [18]. In contrast to the animal manure waste, the food waste could be widely different on the C/N properties due to the waste composition. The Chinese diet habit results in a great proportion of carbohydrate (rice, noodles, and steamed buns) left in the food waste, similar to those food wastes containing high amounts of starches [22], which lead the C/N to be occasionally higher than 20:1. This kind of food waste needs to be recycled by the BSFL, which results in an interesting substrate with high C/N properties and arouses practical questions about what nitrogen source should be used for the nitrogen supplement and what C/N range is suitable for a high performance of BSFL treatment. 

Both inorganic and organic N species could be examined for the nitrogen supplemental effects on the food waste treatment. Previously, biowaste leachate has been reported to be treated by BSFL [23,24]. Since NH_4_-N and NO_3_-N are typical nitrogen species in the leachate, the NH_4_Cl and NaNO_3_ could be tested for the nitrogen supplemental performance. Poultry and swine feces are reported to be effectively degraded by BSFL [20,21]. Since urea and uric acid are typical compounds in the feces, these two chemicals could be tested for the nitrogen supplemental efficiency. BSFL protein is found to be rich with L-Glu and L-Asp amino acids [25]. The L-Glu, L-Asp, as well the simplest amino acid of Gly could be tested for the N supplemental efficiency. Soybean flour and fish meal could be used as positive controls for the organic nitrogen species. As the BSFL are adapted to the poultry manure of C/N 9:1 and less adapted to the cow manure of 20:1 [21,26], the food waste C/N ratio could be adjusted from 20:1 to 10:1 in order to identify the optimal C/N range for a high larval conversion performance.

Therefore, the present study aimed to compare the nitrogen supplying effects of 9 nitrogen species (i.e., NH_4_Cl, NaNO_3_, urea, uric acid, Gly, L-Glu, L-Glu:L-Asp (1:1, *w*/*w*), soybean flour, and fish meal) and further examine the C/N effects on the larval development and bioconversion process for the food waste treatment. Larval enzyme activity and amino acid composition were partially analyzed in order to study the potential physiological effects of nitrogen supplement.

## 2. Materials and Methods

### 2.1. Larvae, Food Waste, and Nitrogen Source Preparation 

Black soldier fly eggs were purchased from a BSF farm (Baiaotai farm, Anyou Biotechnology Group Co., Ltd., Guangxi, China). Upon arrival, eggs were hatched for six days in a substrate containing 60% soybean meal, 30% corn powder, and 10% wheat bran in a 65% moisture content environment at 25 °C. The 6-day old larvae (average weight 0.0027 g) were removed from the hatching substrate through sieving (1 mm mesh) and 45 batches of approximately 800 larvae were weighed. 

Food wastes (FW) were the cooked food leftovers (rice, noodles, vegetables, meats, eggs, etc.) that were collected from the university canteen (Dalian University of Technology, Panjin Campus, Panjin, China). After collection, food wastes were homogenized with a kitchen blender, tested for moisture content in duplicate (oven drying at 105 °C until constant weight [27]), and stored at 4 °C or −20 °C prior to further usage. In Trial 1, 9 nitrogen sources were used, including NH_4_Cl, NaNO_3_, urea, uric acid, Gly, L-Glu, L-Asp, soybean flour, and fish meal, while only urea was used in Trial 2. Within the 9 nitrogen sources, 7 pure compounds with purity grade >99% (NH_4_Cl, NaNO_3_, urea, uric acid, Gly, L-Glu, and L-Asp) were purchased from Aladdin (Shanghai Aladdin Biochemical Technology Co., Ltd., Shanghai, China), and 2 nitrogen sources (soybean flour and fish meal) were purchased from local stores. Carbon and nitrogen contents of the 7 pure compounds were calculated based on their molecular weight, while the C and N properties of the soybean flour, fish meal, as well as the food waste were determined in duplication using the Vario EL cube elemental analyzer (Elementar Analysensysteme GmbH, Hanau, Germany) with the freeze-dried subsamples (Table 1).

### 2.2. Experimental Design

In Trial 1, different nitrogen sources were supplied in 1 g N element per 100 g (dry wt) food waste, i.e., changing N% of food waste from 2.26% to 3.26%. Since the 9 nitrogen sources contained different N and C contents, the actual weights of each nitrogen source used are reported in the Table 1, as well as the C/N values after the nitrogen supplement. In Trial 2, urea was added to the food waste, aiming to adjust food waste C/N from 21:1 (blank control) to 18:1, 16:1, 14:1, 12:1, and 10:1, respectively, while the actual weights of urea used were listed in the Table 1.

All the experiments in Trials 1 and 2 were performed in triplicate, with food waste without nitrogen amendment served as the blank control. Larvae were reared in 4.6 L plastic boxes (240 × 120 × 160 mm) individually. Ten 6 mm diameter holes were made on the box lid in order to enhance passive aeration. In each box, 300 g (wet weight, 70% moisture content) of food waste was added, 150 g food waste was added on Day 0, and another 150 g food waste was added on Day 6. The nitrogen sources were added associated with the food waste according to the amounts in Table 1 on Days 0 and 6, respectively. The 800 weighed larvae (6-days-old) were added into each box on Day 0 following the addition of food waste and nitrogen sources. The boxes were kept at 26–32 °C, and the substrates were mixed manually twice per day. After 12 days, larvae in each box were separated from the frass manually, rinsed with tap water, and dried on paper towels. The total number of larvae was counted. The total wet weight of larvae and frass were recorded, and the moisture content of subsamples of larvae and frass were determined by oven-drying at 105 °C until constant weight [27]. The total dry content of larvae and frass were calculated. Subsamples of larvae were used for the protein and lipid content determination as well as enzyme activity analysis. The rest of the samples were stored at −20 °C for further analysis.

### 2.3. Analysis of Larval Development and Nutrient Composition

Larval length and weight were determined with a two-day interval over 12 days of treatment. Body length was determined in triplicates for larvae within the same box, and the body weight was measured by a combined 10-larva weight that was averaged to obtain the single weight. Larval protein and lipid contents were determined for samples collected on Day 12. To determine protein content, larvae were freeze-dried and milled, larval C and N contents were measured with the Vario EL cube elemental analyzer, and larval protein contents were calculated as the nitrogen content × 4.67 following Janssen et al. [28]. In the lipid analysis, freeze-dried and milled larvae were extracted by petroleum ether (Aladdin) twice (1:10, *w*/*v*, 48 h, 25 °C) in order to achieve crude lipid according to Zheng et al. [29]. After evaporating the petroleum ether, the crude lipid weights were recorded, and the lipid contents were measured as the ratio of crude lipid weight to the larval dry matter. Furthermore, the larval total protein and lipid yield in g/100 g dry waste were evaluated based on the equations below and as mentioned previously [13,30,31].

(1) $$Protein\;yield\;\%\;=\frac{L\times protein\%}{W}\times 100$$

(2) $$Lipid\;yield\;\%=\frac{L\times lipid\%}{W}\times 100$$

where L represents the total dry matter of larvae on Day 12, protein% represents larval protein content (Day 12), lipid% represents larval lipid content (Day 12), and W represents the total dry matter of food waste, including the nitrogen supplement. All parameters were obtained in grams on a dry matter basis. 

### 2.4. Assessment of the Process Efficiency 

To assess the larval treatment efficiency, the larval survival ratio (SR), waste reduction ratio (WR), larvae yield (LY), nitrogen conversion ratio (NCR), efficiency of conversion of digested feed (ECD), as well as the mass distribution pattern were evaluated by applying the aforementioned equations [14,18,30,31]:

(3) $$Survival\;ratio\;\left(SR\right)\;\%\;=\frac{Larvae_{end}}{Larvae_{beg}}\times 100$$

(4) $$Waste\;reduction\;ratio\;\left(WR\right)\;\%=\frac{W-R}{W}\times 100$$

(5) $$Larvae\;yield\;\left(LY\right)\;\%=\frac{L}{W}\times 100$$

(6) $$Nitrogen\;conversion\;ratio\;\left(NCR\right)\;\%=\frac{L\times N{\%}_{larvae}}{W\times N{\%}_{waste}}\times 100$$

(7) $$Efficiency\;of\;conversion\;\left(ECD\right)\;\%=\frac{L}{W-R}\times 100$$

(8) Mass balance: W=R+L+M

where Larvae_beg_ and Larvae_end_ represent the larval numbers at the beginning and end of the treatment respectively, W, R, and L represent the total dry matter of food waste including the nitrogen supplement (W), the frass residue (R), and the larvae (L) respectively, M represents the dry matter loss as a result of larval and microbial metabolism (M), N%_larvae_ represents the nitrogen content of larvae on Day 12, and N%_waste_ represents the nitrogen content of food waste, including the nitrogen supplement. All parameters were obtained in grams on a dry matter basis.

### 2.5. Enzyme Activity and Amino Acid Composition Analysis

Following C/N adjustment with urea in Trial 2, the larval enzyme activity and amino acid composition were further analyzed in triplicate. The activities of antioxidant enzymes, such as peroxidase (POD), superoxide dismutase (SOD), catalase (CAT), and glutathione peroxidase (GSH-px), were observed and recorded. Fresh larvae collected on Day 12 were homogenized by a 1× phosphate-buffered solution (PBS, pH 7.4) in a 1:10 (*w*/*v*) ratio and then centrifuged at 10,000× *g* for 3 min. The supernatants were collected and analyzed for the POD, SOD, CAT, and GSH-px activity following the practice of Chen et al. [32]. Briefly, the POD activity was determined at 420 nm using substrate containing H_2_O_2_, and 1 U of POD was defined as the amount of enzyme that catalyzed 1 μg substrate per liter per minute. The SOD activity was determined at 550 nm through the xanthine and xanthine oxidase system, and 1 U of SOD was defined as the amount of enzyme that created 50% inhibition of xanthine oxidase. The CAT activity was analyzed by measuring the absorbance decrease at 240 nm due to H_2_O_2_ decomposition, and 1 U of CAT was defined as the amount of enzyme that decomposed 1 μmol H_2_O_2_ per liter per second. The GSH-px activity was measured at 423 nm in a system containing 5,5-dithio-bis-(2-nitrobenzoic acid) (DTNB) and reduced GSH, and 1 U of GSH-px was defined as the amount of enzyme that oxidized 1 μmol of reduced GSH per liter per minute. All enzyme activities were calculated and recorded in the unit of U/g larval wet weight.

As for the amino acid composition analysis, freeze-dried and milled larval samples were oxidized by performic acid at 4 °C for 16 h and then hydrolyzed by 6 M HCl with 0.1% phenol at 110 °C for 22 h. The hydrolyzed aliquots were then diluted by 0.02 M HCl and analyzed using a L-8900 High-speed Amino Acid Analyzer (Hitachi High-Tech, Japan) following the instruction manual. For Trp (trytophan), the freeze-dried and milled larval samples were hydrolyzed by 5 M NaOH at 110 °C for 22 h, and the hydrolyzed solutions were tested for Trp through a F-7000 fluorescence detector (Hitachi) with an excitation wavelength of 280 nm and an emission wavelength of 340 nm, as described by Gold et al. [30].

### 2.6. Statistical Analyses

All the statistical analyses were carried out using R 3.4.1 [33]. Differences among groups in the Trials 1 and 2 were tested using analysis of variance (ANOVA) provided by the *multcomp* package [34], which was associated with the *TukeyHSD* function for the pairwise comparison of means. Significance was defined as *p* *<* 0.05.

## 3. Results 

### 3.1. Effects of Nitrogen Source on the Food Waste Treatment

**Figure 1:**
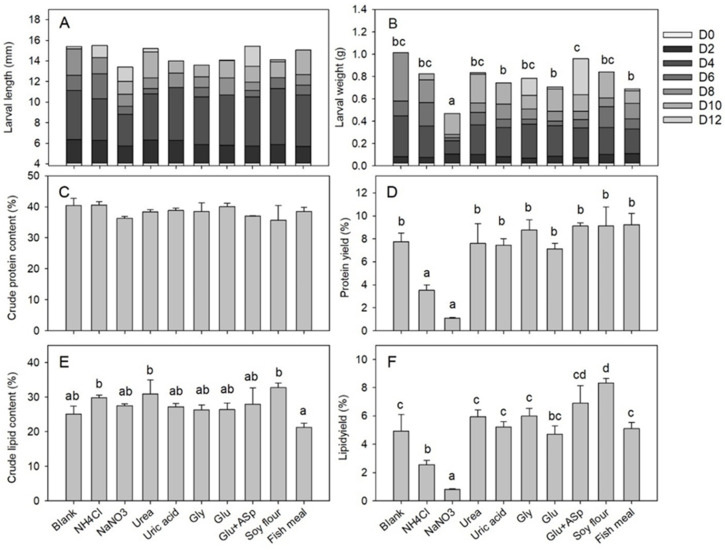
Larval growing and nutrient properties following nitrogen source amendment in Trial 1. Error bars represent standard deviations (*n* = 3). The (**A**) larval body length and (**C**) crude protein content show no significant differences among groups. The (**B**) larval body weight (*p* < 0.001), (**D**) protein yield (*p* < 0.001), (**E**) crude lipid content (*p* = 0.010), and (**F**) lipid yield (*p* < 0.001) are different among groups, and groups with different letters represent significant difference (*p* < 0.05).

Nitrogen source showed limited impacts on the larval length (*df* = 9, *F* = 2.287, *p* = 0.059) and crude protein content (*df* = 9, *F* = 1.398, *p* = 0.253), while in contrast, exerting significant effects on the larval weight (*df* = 9, *F* = 8.167, *p* < 0.001), protein yield (*df* = 9, *F* = 16.410, *p* < 0.001), crude lipid content (*df* = 9, *F* = 3.800, *p* = 0.01), and lipid yield (*df* = 9, *F* = 20.690, *p* < 0.001) as shown in the Figure 1. After 12 days of treatment, the larval body length reached 13.4–15.4 mm (Figure 1A) and crude protein content increased to 36.3–40.6%, regardless of the nitrogen source difference (Figure 1C). For the larval weight, the NaNO_3_ group showed significantly lighter weight than the other groups, with an average at 0.0423 g when other groups’ averages ranged from 0.0688 to 0.0959 g (Figure 1B). For the protein yield, both NH_4_Cl (3.5%) and NaNO_3_ (1.1%) groups were significantly lower than that of the blank control (7.8%), while the results from other groups (7.1–9.3%) were similar to that of the control (Figure 1D). For the crude lipid content, none of the nitrogen source groups were significantly different from the blank control (25.1%), ranging from 21.3% to 30.9% (Figure 1E). For the lipid yield, results from NH_4_Cl (2.6%) and NaNO_3_ (0.8%) groups were significantly lower than that of the blank control (4.9%), with the result of the soybean flour group (8.3%) significantly higher than that of the control and results from the other groups (4.7–6.9%) similar to that of the control (Figure 1F).

**Figure 2:**
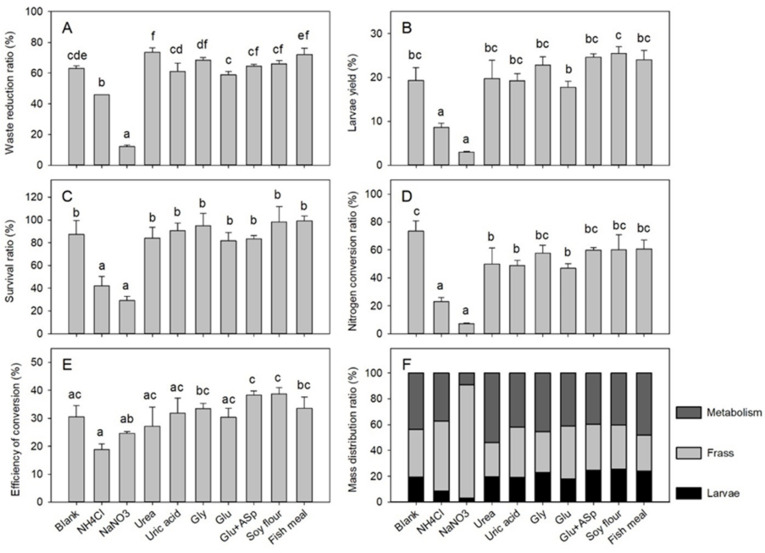
Bioconversion efficiencies following nitrogen source amendment in Trial 1. Error bars represent standard deviations (*n* = 3). The (**A**) waste reduction ratio (*p* < 0.001), (**B**) larvae yield (*p* < 0.001), (**C**) survival ratio (*p* < 0.001), (**D**) nitrogen conversion ratio (*p* < 0.001), and (**E**) efficiency of conversion (*p* = 0.001) are different among groups, and groups with different letters represent significant difference (*p* < 0.05). The (**F**) mass distribution ratio shows no significant differences between groups.

Nitrogen sources substantially affected the process efficiency, as shown by the indexes of WR (*df* = 9, *F* = 90.640, *p* < 0.001), LY (*df* = 9, *F* = 25.080, *p* < 0.001), SR (*df* = 9, *F* = 15.52, *p* < 0.001), NCR (*df* = 9, *F* = 18.88, *p* < 0.001), and ECD (*df* = 9, *F* = 5.438, *p* = 0.001), respectively in the Figure 2. The NH_4_Cl (45.8%) and NaNO_3_ (12.3%) groups displayed significantly lower WR index compared to the blank control (63.1%), whereas the urea group (73.5%) exhibited significantly higher WR than the control, while results from other groups (58.8–72.0%) were similar to that of the control (Figure 2A). The LY and SR indexes exhibited similar trends, that NH_4_Cl (LY 8.6%, SR 42.0%) and NaNO_3_ (LY 3.01%, SR 29.1%) groups were significantly lower than the blank control (LY 19.3%, SR 87.3%), and the other groups (LY 17.8–25.4%, SR 81.7–99.2%) were similar to the control (Figure 2B,C). The NCR index showed that results from the NH_4_Cl (23.1%) and NaNO_3_ (7.2%) groups were significantly lower than that of the control (73.5%), while data from the urea (50.0%), uric acid (48.9%), and Glu (46.8%) groups were higher than that of the NH_4_Cl and NaNO_3_ groups, whereas lower than the control, and data from all other groups (57.7–60.7%) were similar to that of the control (Figure 2D). The ECD indexes varied between 18.9% and 38.7% (Figure 2E), and none of the nitrogen groups demonstrated significantly different ECD compared to the control (30.6%). As for the mass balance analysis, the mass distribution pattern suggested that NH_4_Cl and NaNO_3_ groups resulted in substantially less larvae yield and higher frass residues compared to the blank control, while other nitrogen groups showed approximately similar distribution patterns of larvae, frass, and metabolism mass as the control (Figure 2F).

### 3.2. Effects of C/N on the Food Waste Treatment

**Figure 3:**
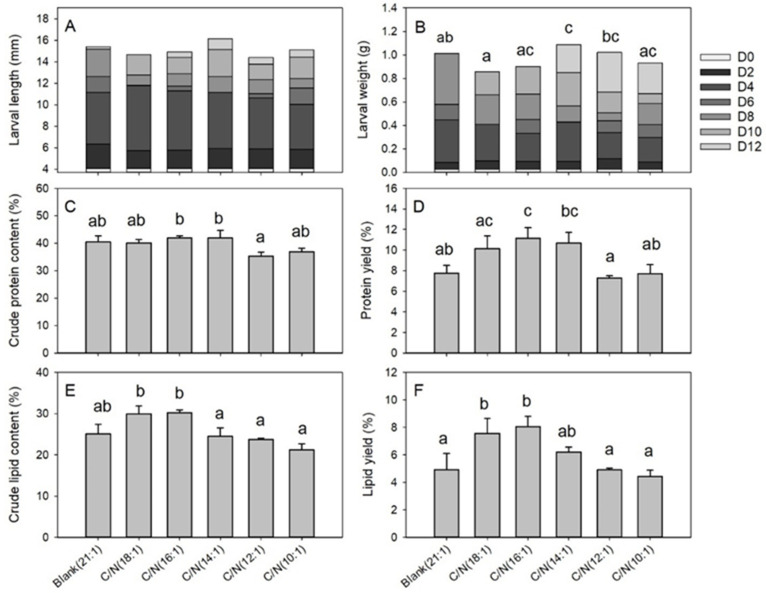
Larval growing and nutrient properties following C/N adjustment in Trial 2. Error bars represent standard deviations (*n* = 3). The (**A**) larval length shows no significant differences between groups. The (**B**) larval weight (*p* = 0.005), (**C**) crude protein content (*p* = 0.011), (**D**) protein yield (*p* = 0.003), (**E**) crude lipid content (*p* = 0.001), and (**F**) lipid yield (*p* = 0.002) are different among groups, and groups with different letters represent significant difference (*p* < 0.05).

The C/N of food waste did not affect larval length (*df* = 5, *F* = 0.488, *p* = 0.779); however, it did influence larval weight (*df* = 5, *F* = 5.934, *p* = 0.005), crude protein content (*df* = 5, *F* = 4.962, *p* = 0.011), protein yield (*df* = 5, *F* = 7.107, *p* = 0.003), crude lipid content (*df* = 5, *F* = 9.874, *p* = 0.001), and lipid yield (*df* = 5, *F* = 8.035, *p* = 0.002) as shown in the Figure 3. Within the 6 C/N groups, larval length ranged from 14.4 to 15.9 mm (Figure 3A). Larval weight varied between 0.074 and 0.109 g (Figure 3B), and the C/N (14:1) group (0.109 g) showed significantly higher body weight than the blank control (0.081 g). Larval crude protein ratio ranged from 35.4% to 42.0%, with none of the 5 C/N group differing from the blank control significantly (Figure 3C). Protein yield ranged from 7.3% to 11.2%, and the C/N (16:1) group (11.2%) yielded significantly higher values than that of the blank control (7.8%) (Figure 3D). Larval crude lipid content changed from 21.2% to 30.3%. None of the 5 C/N groups significantly differed from the blank control, though data from the C/N (18:1–16:1) groups were higher than that of the C/N (14:1–10:1) groups (Figure 3E). Lipid yield varied between 4.4% and 8.1%, with the C/N (18:1) (7.6%) and C/N (16:1) (8.1%) groups exhibiting significantly higher data than the blank control (4.9%) (Figure 3F) and other C/N groups (4.4–6.2%), demonstrating results similar to the control. 

**Figure 4:**
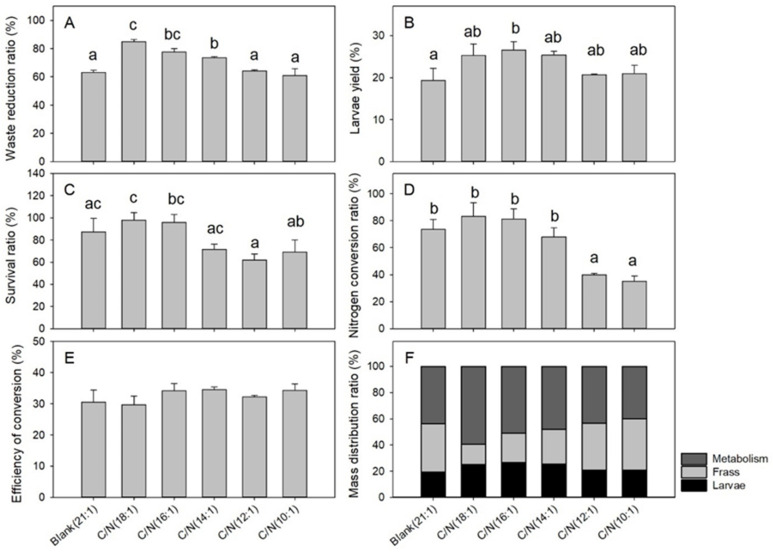
Bioconversion efficiencies following C/N adjustment in Trial 2. Error bars represent standard deviations (*n* = 3). The (**A**) waste reduction ratio (*p* < 0.001), (**B**) larvae yield (*p* = 0.015), (**C**) survival ratio (*p* = 0.004), and (**D**) nitrogen conversion ratio (*p* < 0.001) are different among groups, and groups with different letters represent significant difference (*p* < 0.05). The (**E**) efficiency of conversion and (**F**) mass distribution ratio show no significant differences among groups.

The C/N of food waste greatly affected the process efficiency, as indicated by the WR (*df* = 5, *F* = 29.630, *p* < 0.001), LY (*df* = 5, *F* = 4.540, *p* = 0.015), SR (*df* = 5, *F* = 6.468, *p* = 0.004), and NCR (*df* = 5, *F* = 19.400, *p* < 0.001) indexes in the Figure 4. However, the ECD (*df* = 5, *F* = 1.583, *p* = 0.238) index was less affected. The WR indexes ranged from 61.0% to 84.8%, and the C/N (18:1) (84.8%), C/N (16:1) (77.6%), and C/N (14:1) (73.5%) groups yielded significantly higher data than that of the blank control (63.1%) (Figure 4A). The LY indexes changed from 19.3% to 26.6%, and the C/N (16:1) (26.6%) group was significantly higher than the blank control (19.3%), while the other 4 C/N groups ranged from 20.6% to 25.4% of BCRs (Figure 4B). The SR indexes changed from 61.9% to 98.0%, and none of the 5 C/N groups differed from the blank control significantly (Figure 4C). The NCR indexes showed a pattern which indicated that the C/N (18:1) (83.0%), C/N (16:1) (81.3%), and C/N (14:1) (68.1%) groups were similar to the blank control (73.5%), while the C/N (12:1) (39.9%) and C/N (10:1) (35.2%) groups were lower than the control (Figure 4D). The ECD indexes varied within 29.7% and 34.6% and none of the 5 C/N groups differed significantly from the blank control (30.6%) (Figure 4E). The mass balance analysis suggested that the C/N (18:1), C/N (16:1), and C/N (14:1) groups together resulted in relatively higher larval ratios and lower frass ratios compared to the blank control, C/N (12:1), and C/N (10:1) groups (Figure 4F).

### 3.3. Effects of C/N on Larval Enzyme Activity and Amino Acid Composition 

Among all the four larval enzymes tested, only the SOD activity (*df* = 5, *F* = 4.561, *p* = 0.015) differed greatly between the 6 C/N groups as shown in the Table 2, while the POD (*df* = 5, *F* = 2.266, *p* = 0.114), CAT (*df* = 5, *F* = 1.710, *p* = 0.207), and GSH-px (*df* = 5, *F* = 0.816, *p* = 0.561) activities did not. For the amino acid composition analysis, the compositions of each amino acid among the 6 C/N groups were not significantly different as shown in the Table 3. The averaged proportions of each amino acid were therefore calculated, and the Glu, Ala, and Asp were found to be the top 3 most abundant amino acids.

## 4. Discussion

### 4.1. Effects of Nitrogen Source on the Bioconversion Process

Among all the 9 nitrogen sources, the NH_4_Cl and NaNO_3_ resulted in markedly adverse effects on the larval development and process efficiency compared to the 7 other organic nitrogen species, suggesting that NH_4_Cl and NaNO_3_ were probably less suitable than the organic nitrogen species in terms of facilitating larval development and waste degradation. The less efficient performance in the NH_4_Cl and NaNO_3_ conditions were probably due to several reasons, including: (1) the high amount of compounds used, (2) the toxicity generated by chloride or sodium salt, and (3) the low survival and adaptability of BSFL to these environments. In general, NH_4_-N and NO_3_-N were typical nitrogen species in the leachate and sludge biowaste. Larvae that fed on liquid leachate [24] and sewage sludge [18] have been found to be of low survival rate (30–40% mortality) and low bioconversion performance (LY 0.2–2.3%), which complied with the present results in the NH_4_Cl and NaNO_3_ conditions. However, Green et al. [23] reported that feeding BSFL with 10 mM NaNO_3_ solution, specifically 14 mg NO_3_-N/100 mL solution, facilitated the BSFL’s transformation of NO_3_-N to NO_2_-N and further to NH_4_-N (e.g., denitrification). The current study supplied 1 g NH_4_-N or NO_3_-N/100 g dry matter to the food waste. The high amount of N element could be one of the main reasons for the negative effects of NH_4_Cl and NaNO_3_ effects on the BSFL compared to Green’s study. However, as a nitrogen source used for nitrogen amendment of food waste, addition of 1 g N/100 g dry matter is a reasonable requirement that NH_4_-N and NO_3_-N might fail to address the need due to their negative effects on BSFL.

Interestingly, a subpopulation of larvae survived after the NH_4_Cl and NaNO_3_ amendments in the study. This subpopulation resulted in a similar protein and lipid body ratio when compared with the larvae which grew in the 7 other organic nitrogen conditions, suggesting that a small percentage of BSFL may gain the ability to adapt to NH_4_Cl and NaNO_3_ nutrient/environment, either through the direct incorporation of the NH_4_-N/NO_3_-N or through the indirect utilization of NH_4_-N/NO_3_-N assimilated by the in vivo or in vitro microorganisms. Barragan-Fonseca et al. [35] has pointed out that the larval protein content is regulated within narrow constraints, whereas the fat content is strongly impacted by nutrient concentration. The present results agreed with these findings that larval protein content was limitedly affected by the nitrogen environment in terms of BSFL survival; however, the larval lipid content was significantly affected by the nitrogen sources, where urea, L-Glu/L-Asp, and soybean flour were probably better nutrient sources for BSFL compared to other nitrogen species due to the relatively higher lipid content or lipid yield of BSFL.

Food waste amended with the 7 organic nitrogen sources generally resulted in neither negative nor positive effects on the BSFL performance compared to the control based on the bioconversion indexes of WR, LY, SR, and ECD. One of the possible reasons could be the inefficient supplemental ratio of the organic nitrogen sources to the food waste. According to the NCR index, all the nitrogen amendment conditions were lower than the control, suggesting that the optimum nitrogen supplying amount was not achieved, and the NCR values (46.8–60.7%) of the 7 organic nitrogen conditions were less than a previous study (66.4% ± 6.5%) conducted for mussel and bread waste treatment [13]. These results suggest that the addition amount of selected nitrogen source is very important and should be critically optimized in order to achieve high performance of waste reduction and BSFL bioconversion efficiency.

### 4.2. Effects of C/N on the Bioconversion Process

Although there is no well-known regulation about whether urea could be used as a food additive for the BSFL, urea is a suitable nitrogen source for investigating the C/N effects on the food waste treatment by the BSFL. The reasons are as follow: (1) Urea is a nitrogen source containing the highest N content (46.7%) and lowest C content (20.0%) amongst the 7 organic nitrogen species, which allow urea to be one of the most efficient nitrogen sources used for nitrogen content amendment while simultaneously limiting the energy/nutrient effect generated by the carbon. (2) Urea exhibited a feasible nutrient effect to BSFL according to the lipid and protein analysis in the Trial 1, (3) urea is a natural food source for BSFL as it is contained in the animal feces [19,21], and (4) urea is widely available as an artificially synthesized chemical. Therefore, urea was selected as the nitrogen source used for C/N adjustment in the Trial 2 of this study.

The larval protein and lipid data suggested that the C/N range of 18:1 to 16:1 was optimal for a high larval protein and lipid yield, while the bioconversion indexes indicated that the C/N range of 18:1 to 14:1 was highly efficient for the waste reduction (WR, 73.5–84.8%, *p* < 0.001) and larvae production (LY, 10.1–11.1%, *p* = 0.003). Therefore, supplying food waste with a moderate amount of urea (adjusting food waste C/N from 21:1 down to 18:1–14:1, especially 18:1–16:1) significantly facilitated larval development and food waste consumption; however, further addition of urea (lowering the C/N down to 12:1–10:1) would result in urea waste and even negative effects on the larval growing and bioconversion process, as indicated by the declining WR, LY, and NCR indexes.

The current results suggest that overdosing nitrogen-rich material would result in larval mortality and declined process efficiency. Similar results have also been seen in two other recent studies. Lopes et al. [14] studied recycling aquaculture waste by feeding BSFL with fish waste and bread mixture, and Ewald et al. [13] has tried to manipulate larval fatty acid composition by feeding BSFL with mussel and bread mixture. Both studies suggested that adding a moderate amount of nitrogen-rich aquaculture waste was beneficial for larval development, while too much nitrogen material may lead to negative effects such as larval mortality and biomass loss. Based on the larvae yield, this moderate range for fish waste treatment was approximately 5–15% of fish carcasses [14], and approximately 10–20% of mussel for the mussel material [13]. Unfortunately, neither studies reported the C or N content of the diet materials. If roughly assuming the bread C and N content [36] to be 48.9% of C (50% of organic matter) and 2.95% of N (16% of crude protein), and fish C and N content to be 45% of C and 66% of N (as fish meal in this study), the 5–15% of fish waste in Lopes’ study is thus equivalent to C/N of 15:1–12:1, which is close to the optimum range of 18:1–14:1 found in this study, indicating the beneficial effects of modifying C/N of food waste into this range. As for the applying amount of nitrogen amendment, urea could be more efficient than aquaculture waste, as 0.36–1.10 g urea/100 g dry matter (equivalent to 0.11–0.33% wet weight basis) used in this study resulted in comparable performance improvements compared with the 5–15% of fish waste [14] or 10–20% of mussel waste [13] used in previous studies.

Interestingly, the C/N adjustment through urea altered the larval production performance but not the crude protein content or the amino acid composition. In the fish and mussel studies [13,14], a clear trend of the positive correlations between the aquaculture materials and larval protein content was observed, although higher larval mortality occurred simultaneously with more aquaculture waste used. This finding suggests that the urea may not support the BSFL growth directly with the amino acid nutrient, whereas it may improve larval development through modulating larval metabolism, such as the SOD enzyme activity. Interestingly, the amino acid composition of BSFL is relatively stable despite wide variations of C/Ns. This could be highly due to the same nitrogen source, i.e., urea, used in the current C/N trial. In another study where BSFL fed on different substrates [18], the amino acid composition among groups varied greatly, and Tyr, Glu, and Asp were found as the top 3 amino acids for BSFL fed on human feces, and Glu, Asp, and Lys were found as the top 3 species for the food waste substrates. The current study also found Glu and Asp as the top 2 amino acids, whereas the third abundant species was Ala. Compared to the previous study [18], the Cys, Met, Thr, Ser, Gly, and Pro proportions were generally higher, and the Tyr and Lys proportions were generally lower in the larvae of current study. These results suggest that the nitrogen species may influence BSFL amino acid composition more greatly than the C/N ratios.

Altogether, the waste reduction performances of 73.5–84.8% and larvae yield of 25.3–26.6% at the C/N conditions of 18:1–14:1 in the current study are higher than many of the previous BSFL studies [3,19]. Other than the higher nutrient and digestibility of food waste used in current study, the nitrogen supplement of urea and optimal range of C/N could be two of the main contributors to the performance improvement.

## 5. Conclusions

Adjusting the C/N of food waste substrate is a viable method for improving the larval treatment performance. Organic nitrogen is more suitable than the NH_4_Cl or NaNO_3_ as the nitrogen amendment. Urea was a reliable and practical nitrogen source for the C/N adjustment. Addition of small amounts of urea, targeting C/N of 18:1–14:1, may significantly improve the waste reduction performance, while targeting C/N of 18:1–16:1 may substantially increase the larval protein and lipid conversion efficiency, and the BSFL amino acid composition was not affected by the C/N variation. Therefore, the current study reveals that the C/N range of 18:1–16:1 is likely the optimal condition for food waste treatment by BSFL, and the application of the current strategy may improve the food waste biodegradation and facilitate the nutrient recycling by the BSFL farming.

## Figures and Tables

**Table 1:** Experimental design of nitrogen source supplied in Trials 1 and 2.

Groups	N Source	N Element	N Source	Final Carbon and Nitrogen Properties		
	Supplied ^1^	Amount	Amount	after Nitrogen Source Supplement		
		g/100 g FW	g/100 g FW	C (%)	N (%)	C/N
Trial 1						
Blank	None	0	0	47.0	2.26	21:1
NH_4_CL	NH_4_CL	1	3.82	47.0	3.26	14:1
NaNO_3_	NaNO_3_	1	6.06	47.0	3.26	14:1
Urea	Urea	1	2.14	47.4	3.26	15:1
Uric acid	Uric acid	1	3.00	48.1	3.26	15:1
Gly	Gly	1	5.35	48.7	3.26	15:1
Glu	L-Glu	1	10.53	51.3	3.26	16:1
Glu/Asp	L-Glu/L-Asp (1:1)	1	10.00	50.9	3.26	16:1
Soybean flour	Soybean flour	1	16.72	55.1	3.26	17:1
Fish meal	Fish meal	1	9.52	50.8	3.26	16:1
Trial 2						
Blank (21:1)	None	0	0	47.0	2.26	21:1
C/N(18:1)	Urea	0.36	0.76	47.2	2.62	18:1
C/N(16:1)	Urea	0.68	1.46	47.3	2.94	16:1
C/N(14:1)	Urea	1.10	2.36	47.5	3.36	14:1
C/N(12:1)	Urea	1.66	3.55	47.7	3.92	12:1
C/N(10:1)	Urea	2.44	5.23	48.0	4.70	10:1

^1^ The C and N contents were determined for food waste (FW, C 47.0%, N 2.26%, *n* = 2), soybean flour (C 48.5%, N 5.98%, *n* = 2), and fish meal (C 39.9%, N 10.5%, *n* = 2) materials. All parameters were obtained on a dry matter basis.

**Table 2:** Activities of antioxidant enzyme of larvae collected on Day 12 in Trial 2.

Groups	POD (U/g)	SOD (U/g)	CAT (U/g)	GSH-px (U/g)
Blank (21:1)	0.056 ± 0.079	0.149 ± 0.082 ^ab^	0.071 ± 0.034	7.40 ± 0.78
C/N (18:1)	0.00 ± 0.00	0.249 ± 0.081 ^b^	0.094 ± 0.026	8.46 ± 1.77
C/N (16:1)	0.049 ± 0.048	0.213 ± 0.133 ^ab^	0.035 ± 0.034	9.91 ± 4.56
C/N (14:1)	0.00 ± 0.00	0.058 ± 0.009 ^ab^	0.039 ± 0.027	7.12 ± 2.03
C/N (12:1)	0.170 ± 0.121	0.00 ± 0.00 ^a^	0.037 ± 0.018	4.90 ± 0.62
C/N (10:1)	0.00 ± 0.00	0.00 ± 0.00 ^a^	0.042 ± 0.008	5.94 ± 1.39
	*F* = 2.266, *p* = 0.114	*F* = 4.561, *p* = 0.015	*F* = 1.710, *p* = 0.207	*F* = 0.816, *p* = 0.561

Values are presented as mean ± standard deviation (*n* = 3). Different letters represent significant differences among column-wise groups.

**Table 3:** Proximate amino acid compositions of larvae collected on Day 12 in Trial 2.

Amino Acids	Blank (21:1)	C/N (18:1)	C/N (16:1)	C/N (14:1)	C/N (12:1)	C/N (10:1)	Overall
Cys	1.0 ± 0.1	0.9 ± 0.1	0.8 ± 0.0	0.9 ± 0.0	0.8 ± 0.0	0.8 ± 0.0	0.9 ± 0.1
Met	3.3 ± 0.2	3.4 ± 0.1	3.2 ± 0.1	3.3 ± 0.2	3.2 ± 0.2	3.1 ± 0.1	3.2 ± 0.2
Asp	8.5 ± 0.4	9.2 ± 1.0	9.1 ± 0.8	8.8 ± 0.3	9.4 ± 0.8	9.5 ± 0.6	9.1 ± 0.8
Thr	4.4 ± 0.2	4.5 ± 0.3	4.5 ± 0.2	4.3 ± 0.2	4.5 ± 0.2	4.5 ± 0.2	4.4 ± 0.2
Ser	5.0 ± 0.2	5.0 ± 0.3	5.1 ± 0.3	5.0 ± 0.2	4.9 ± 0.2	5.0 ± 0.2	5.0 ± 0.3
Glu	10.6 ± 0.5	11.2 ± 0.7	11.6 ± 0.3	11.7 ± 1.2	10.8 ± 0.6	12.3 ± 0.5	11.4 ± 0.9
Gly	6.2 ± 0.3	6.0 ± 0.2	6.2 ± 0.2	6.1 ± 0.3	5.9 ± 0.3	5.8 ± 0.2	6.0 ± 0.3
Ala	11.1 ± 0.3	10.2 ± 0.5	9.9 ± 0.6	10.0 ± 0.8	10.0 ± 0.2	9.1 ± 0.3	10.0 ± 0.8
Val	7.0 ± 0.5	6.9 ± 0.5	7.0 ± 0.3	6.7 ± 0.2	6.8 ± 0.5	6.4 ± 0.4	6.8 ± 0.4
Ile	4.9 ± 0.3	4.8 ± 0.3	4.8 ± 0.2	4.8 ± 0.2	4.9 ± 0.3	4.8 ± 0.3	4.8 ± 0.3
Leu	7.5 ± 0.2	7.5 ± 0.5	7.5 ± 0.3	7.5 ± 0.1	7.9 ± 0.4	7.8 ± 0.1	7.6 ± 0.3
Tyr	0.7 ± 0.1	0.6 ± 0.1	0.5 ± 0.2	0.5 ± 0.0	0.6 ± 0.1	0.5 ± 0.0	0.6 ± 0.1
Phe	4.5 ± 0.5	4.7 ± 0.4	4.6 ± 0.1	4.4 ± 0.2	4.7 ± 0.5	4.6 ± 0.3	4.6 ± 0.4
Lys	5.9 ± 0.2	5.9 ± 0.1	6.1 ± 0.2	6.0 ± 0.2	6.3 ± 0.1	6.4 ± 0.2	6.1 ± 0.3
His	3.5 ± 0.2	3.6 ± 0.2	3.5 ± 0.1	3.5 ± 0.1	3.3 ± 0.2	3.1 ± 0.1	3.4 ± 0.2
Arg	5.2 ± 0.1	4.9 ± 0.5	4.8 ± 0.4	5.2 ± 0.0	5.0 ± 0.1	5.3 ± 0.1	5.1 ± 0.3
Pro	7.3 ± 0.1	7.1 ± 0.3	7.0 ± 1.1	7.5 ± 0.4	7.2 ± 0.1	7.5 ± 0.4	7.3 ± 0.5
Trp	1.3 ± 0.4	1.6 ± 0.4	1.7 ± 0.1	1.6 ± 0.4	1.9 ± 0.1	1.5 ± 0.0	1.6 ± 0.3

Values are presented as g/100 g protein with mean ± standard deviation (*n* = 3).

## Data Availability

The data presented in this study are openly available in FigShare at doi.10.6084/m9.figshare.14609535.
